# Hepatocyte Injury and Hepatic Stem Cell Niche in the Progression of Non-Alcoholic Steatohepatitis

**DOI:** 10.3390/cells9030590

**Published:** 2020-03-02

**Authors:** Diletta Overi, Guido Carpino, Antonio Franchitto, Paolo Onori, Eugenio Gaudio

**Affiliations:** 1 Department of Anatomical, Histological, Forensic Medicine and Orthopedics Sciences, Sapienza University of Rome, 00161 Rome, Italy; diletta.overi@uniroma1.it (D.O.); antonio.franchitto@uniroma1.it (A.F.); paolo.onori@uniroma1.it (P.O.); 2 Department of Movement, Human and Health Sciences, Division of Health Sciences, University of Rome “Foro Italico”, 00135 Rome, Italy; guido.carpino@uniroma1.it

**Keywords:** liver, progenitor cell, regeneration, macrophage, disease, fibrosis, lipotoxicity, adipose tissue, atherosclerosis, ductular reaction

## Abstract

Non-alcoholic fatty liver disease (NAFLD) is a chronic liver disease characterized by lipid accumulation in hepatocytes in the absence of excessive alcohol consumption. The global prevalence of NAFLD is constantly increasing. NAFLD is a disease spectrum comprising distinct stages with different prognoses. Non-alcoholic steatohepatitis (NASH) is a progressive condition, characterized by liver inflammation and hepatocyte ballooning, with or without fibrosis. The natural history of NAFLD is negatively influenced by NASH onset and by the progression towards advanced fibrosis. Pathogenetic mechanisms and cellular interactions leading to NASH and fibrosis involve hepatocytes, liver macrophages, myofibroblast cell subpopulations, and the resident progenitor cell niche. These cells are implied in the regenerative trajectories following liver injury, and impairment or perturbation of these mechanisms could lead to NASH and fibrosis. Recent evidence underlines the contribution of extra-hepatic organs/tissues (e.g., gut, adipose tissue) in influencing NASH development by interacting with hepatic cells through various molecular pathways. The present review aims to summarize the role of hepatic parenchymal and non-parenchymal cells, their mutual influence, and the possible interactions with extra-hepatic tissues and organs in the pathogenesis of NAFLD.

## 1. Introduction

Non-alcoholic fatty liver disease (NAFLD) is a chronic liver disease characterized by hepatic fat accumulation in the absence of excessive alcohol consumption, and defined by the presence of steatosis in at least 5% of hepatocytes [1]. NAFLD is a heterogeneous disease, comprising distinct histological conditions with different prognoses [1]. Non-alcoholic fatty liver (NAFL) is defined as the presence of hepatic steatosis in at least 5% of the hepatocytes, without evidence of hepatocellular injury in the form of hepatocyte ballooning; non-alcoholic steatohepatitis (NASH) is defined as the presence of at least 5% hepatic steatosis and inflammation with hepatocyte injury (e.g., ballooning), with or without fibrosis [2]. The term NASH covers a wide spectrum of disease severity, including progressive fibrosis and cirrhosis. Remarkably, both NAFL and NASH can cause hepatocellular carcinoma (HCC) in the presence or absence of liver fibrosis and cirrhosis; in these patients, HCC incidence can vary from 2.4% to 12.8% [3].

The global prevalence of NAFLD is currently estimated to be 24%, and it is highly spread in all continents [4]. The prevalence of NAFLD is constantly increasing and, similarly, the rate of NASH has almost doubled in the past years; moreover, NASH is now considered the second most common indication for liver transplantation in the USA [4]. Both NAFL and NASH are becoming increasingly prevalent as the epidemics of obesity and diabetes continue to increase. A mathematical model was built to understand how the disease burden associated with NAFLD and NASH will change over time, and the results suggest an increase in the number of cases of advanced liver disease and in liver-related mortality in the coming years, in concert with a global pandemic of obesity [5]. From a clinical perspective, NAFLD is associated with cardiovascular disease, and the two disorders share several cardio-metabolic risk factors [2,6]. NAFLD represents an important issue in the pediatric population, representing the leading cause of chronic liver disease in adolescents and young adults. The prevalence of children obesity is increasing in most regions of the world [7,8], causing a rise in the risk of developing chronic diseases, such as type 2 diabetes, cardiovascular disease, and NAFLD [9].

From an epidemiological and clinical perspective, the increased cardio-metabolic [2] and tumorigenic [3] risk in NAFLD patients seems to depend strongly on the presence of advanced stages of NAFLD, such as NASH, with moderate-to-advanced fibrosis; therefore, basic and translational sciences are making efforts to individuate pathogenetic mechanisms and cellular cross-talks at the basis of NASH evolution and fibrosis development. The present review aims to summarize the role of hepatic parenchymal and non-parenchymal cells and their cross-talks in the pathogenesis of NAFLD, and the possible interactions with extra-hepatic tissues/organs.

## 2. Hepatocyte Damage in NAFLD

### 2.1. Hepatocytes in Physiological Turnover and Regeneration

Hepatocytes represent a cellular population characterized by high proliferative capabilities, which support the physiological renewal of liver parenchyma [10]. Definite subsets of hepatocytes located in a precise position within the liver lobule have been described as main actors in liver homeostasis and regeneration. Around the centrilobular vein, subpopulations of diploid Axin2^+^ [11] and Lgr5^+^ [12] hepatocytes have been individuated; both these subpopulations are characterized by self-renewal properties and their progeny, during homeostasis, can generate pericentral hepatocytes. However, the role of these subpopulations in generating periportal hepatocytes is controversial [13,14]. In fact, at periportal zone, hepatocyte subpopulation expressing Sox9 [15] or Mfsd2a [16] were identified and individuated as major contributors in the regeneration of zone 1 hepatocytes during injury-induced regeneration.

Recently, a rare subset of hepatocytes that expresses high levels of telomerase and distributed throughout the liver lobule were demonstrated to be able to regenerate hepatocytes in all lobular zones [10]. Similarly, recent evidence have further disclosed the dynamics of hepatocyte replication in physiological turnover and in regeneration after injury, demonstrating that most hepatocytes throughout the lobule participate in maintaining the hepatocyte mass and proliferate to regenerate it, with diploid cells holding a growth advantage over polyploid ones [12,13,17,18].

### 2.2. Morphological Alterations in Hepatocytes

**Figure 1:**
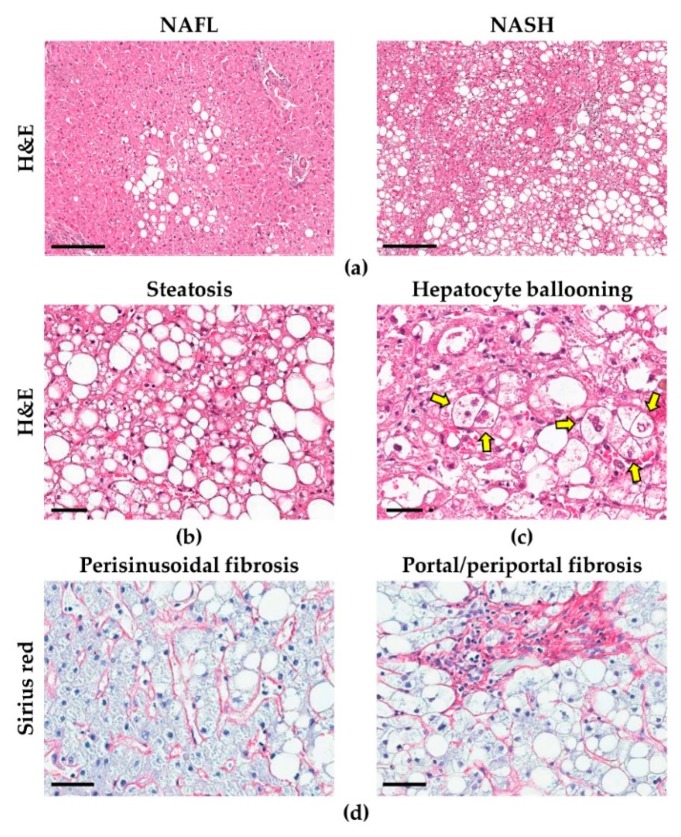
Histo-morphological features of non-alcoholic fatty liver disease (NAFLD). The progression from simple steatosis (non-alcoholic fatty liver—NAFL) to non-alcoholic steatohepatitis (NASH) (**a**) is characterized by increased hepatic steatosis (**b**) and inflammation, accompanied by the emergence of specific histological features, such as hepatocellular ballooning (arrows in **c**). As disease advances, liver fibrosis develops (**d**). H&E: hematoxylin and eosin; scale bars: 200 (**a**), 50 (**b**,**c**) and 100 μm (**d**). Images obtained from liver biopsies of patients affected by NAFLD.

The morphological hallmark of NAFLD is the presence of hepatic steatosis, i.e., the accumulation of fat within the hepatocytes (Figure 1) [19,20]. In NAFLD patients, usually, large fat droplets (i.e., macrovesicular steatosis) are observed inside the hepatocytes but, occasionally, smaller areas of microvesicular steatosis can be found [19]. Pericentral hepatocytes, compared to periportal ones, are the most subjected to steatosis, due to their specific role in fat metabolism [20]; as a consequence, in early phases of NAFLD, hepatic steatosis is mainly located around the centrilobular vein, extending towards portal tracts as the entity of steatosis increases and hepatic zonation is lost [19,20]. The continuous exposure of hepatocytes to cellular stressors leads to the emergence of specific histological features of NASH, such as hepatocellular ballooning and Mallory-Denk Bodies (MDBs, or Mallory’s hyaline), which also represent negative prognostic indexes [19,21]. Ballooned hepatocytes are larger than normal ones, and are characterized by rarified, irregular cytoplasm, and by the loss of positivity for cytokeratins (CK) 8 and 18 [19,22]. MDBs are eosinophil accumulations of ubiquitinated proteins within the cytoplasm of hepatocytes, and can be identified in routine stains (especially in ballooned hepatocytes) or highlighted by immunohistochemistry for bound proteins (i.e., ubiquitin or p62) [19].

### 2.3. Lipotoxicity in Hepatocytes

Lipotoxicity is considered the cellular damage due to the accumulation of abnormal lipid compounds in the cell, leading to the formation of reactive species of oxygen (ROS) [22,23]. NAFLD patients are characterized by an increased load of free fatty acids (FFAs) in the liver, which can be due both to increased lipolysis from adipose tissue but also to de novo lipogenesis in hepatocytes [24,25,26,27,28,29,30]. Insulin resistance has a prominent role in these processes by favoring an increased lipolytic response to the meal, and by inducing the expression of lipogenic pathways in the liver [24,25,27,31,32]. In the liver, FFAs are metabolized by beta-oxidation in mitochondria, or esterified as triglycerides (TGs), and either secreted within very-low-density lipoproteins (VLDL) or stored in lipid droplets leading to hepatic steatosis [25]. With the progression toward NASH, hepatocytes become increasingly sensitive to damage and incapable to respond to injury due to the accumulation of toxic lipid metabolites, the production of ROS, and the dysfunction of detoxification responses [23,26]; in parallel, VLDL lipolysis and production are decreased, leading to further accumulation of TGs in hepatocytes [33,34]. One of the main effectors of damage-induced response is c-Jun N-terminal kinase (JNK). JNK is a member of the mitogen-activated protein kinase (MAPK) family and represents the downstream effector for several signaling pathways leading to an increased expression of pro-apoptotic and pro-inflammatory transcription factors [25,35]. NASH patients are characterized by increased phosphorylation (i.e., activation) of JNK [23,36,37], which can be due both to a direct effect of FFAs, or to the activation of nuclear factor-κB (NF-κB) pathway [26,38]. Upregulation of JNK pathway also leads to inactivation of insulin receptor, aggravating insulin resistance in hepatocytes [24,26].

Genome-wide studies have been able to identify genetic determinants of NAFLD. Among these, the single nucleotide polymorphism in residue 148 (I148M, rs738409) in human patatin-like phospholipase domain containing 3 (PNPLA3) gene, encoding the protein adiponutrin, has been recognized as one of the strongest genetic factors leading to NAFLD development [39,40]. Interestingly, the relationship between PNPLA3 variant and NAFLD development was independent to metabolic risk factors and lipid profile [40]. Although the basis of this association has not been fully elucidated, PNPLA3 variant carriers are characterized by reduced hydrolasic activity of adiponutrin, leading to increased lipid content in the liver [41,42]. Interestingly, PNPLA3 I148M carriers are characterized by worse histological depicts, with steatosis occurring in periportal hepatocytes also in early-grade disease [43,44,45].

### 2.4. Endoplasmic Reticulum Stress and Mitochondrial Dysfunction in NAFLD

Normal hepatocytes are characterized by an extensive endoplasmic reticulum (ER), and this organelle can be severely affected in course of chronic metabolic unbalance and cellular stress [28,46,47,48,49]. De novo lipogenesis occurs in ER and is regulated by membrane proteins sterol regulatory element-binding proteins (SREPB1c and SREPB2, for fatty acids and cholesterol respectively) and related pathways [24,25,38,46]. In presence of insulin resistance, these proteins are upregulated, leading to increased lipogenesis and further lipotoxicity [24,25,28,38,50]. Moreover, the hepatic accumulation of fat can lead to altered composition of ER membrane, leading to impaired functionality [46,51,52].

All membrane and secreted proteins (e.g., lipoproteins) are synthesized and/or assembled on the ER, which represents a highly active task in the hepatocyte; in this context, injured hepatocytes are characterized by an increase in misfolded proteins which accumulate in the cytoplasm (e.g., MDBs), can overload the ER and, subsequently, trigger the so-called unfolded protein response (UPR), a protective pathway which is aimed to reduce damage to the cell; however, when extensive or chronic damage occur, this response can be overwhelmed and, in turn, lead to cell death [24,46,53]. ER is endowed with stress sensors that respond to injury signals leading to UPR activation; among these, the transmembrane protein inositol-requiring enzyme 1 α (IRE1α) plays a crucial role, interacting with different pathways in the cell [54]. By binding to misfolded proteins or lipids, it can phosphorylate JNK and IκB (upstream of NF-κB pathway), leading to reduced insulin sensitivity and pro-inflammatory pathway activation [24,38,55]. Moreover, ER stress can lead to increased inflammasome pathway activation and further hepatocyte injury, eventually leading to a shift towards pro-apoptotic signaling pathways [24,28,48,56,57,58,59,60].

Hepatocytes are characterized by a high number of mitochondria. Under normal conditions, mitochondria are the major site of ROS formation in the cell, with ~2% of consumed O_2_ converted in ROS [61,62]. Moreover, mitochondria can also furnish intracellular signals leading to adaptation of the cell to the environment [61]: in the first stages of NAFLD, mitochondria increase their activity in response to the rising lipid levels in the hepatocytes, with a protective effect [23,25]. In this context, the exposure to oxidative stress triggers the adaptation of mitochondria (i.e., mitochondrial remodeling), with morphological modifications occurring through mitochondrial fission and fusion, and with variations in energy expenditure and gene expression [63].

According to these observations, mitochondria undergo pathological modifications in course of NAFLD, especially when progressing towards NASH, with impairment in adaptive capabilities, reduced ATP production and increased oxidative stress in the cell [24,25,35,63,64,65,66,67,68]. Moreover, ultrastructural damage to the mitochondria characterizes liver biopsies from NASH patients [69,70]. In particular, damaged hepatocytes show the presence of enlarged mitochondria, characterized by the loss of cristae and by the presence of crystalline inclusions [66,70,71]; in some cases, megamitochondria (3–10 μm in diameter) can be found, being also visible in Masson trichrome stain as red inclusions within the hepatocytes [19,72,73]. The formation of megamitochondria likely involves unbalanced mitochondrial division and fusion, and recent data in rodent NASH models indicated that extreme mitochondrial size contributes to hepatocyte dysfunction [74]; moreover, the increased number of mitochondria observed in NASH seems to be due mainly to defects in the removal of damaged organelles via autophagy (in this case, mitophagy) than to increased mitochondrial biogenesis [23,25,60]. Several mechanisms might be involved in mitophagy alteration in NAFLD [75], such as the impairment of a parkin-independent mitophagy pathway, based on p62-regulated mitochondrial ubiquitination by Keap1 and Rbx1 [74].

In NAFLD patients, products of lipid metabolism lead to damage to mtDNA and mitochondrial respiratory chain (MRC) proteins [23,25,67,74]; moreover, the binding of activated JNK to MRC complexes leads to increased ROS formation [25,35]. This aspect is particularly evident in the progression towards NASH, were increased ROS release by mitochondria is accompanied by reduced catalase activity, leading to impaired detoxification and further damage to the organelle [23,25,76,77]. Moreover, excess cholesterol can lead to a loss of glutathione by mitochondria, aggravating the reduced state of the cell [38] and leading to altered beta-oxidation and lipotoxicity [24]. Finally, hepatocyte necrosis could lead to the release of mitochondria-derived danger associated molecular patterns (DAMPs), which in turn could activate NLRP3 (NACHT, LRR and PYD domains-containing protein 3) inflammasome pathway (see also the following section) [78,79,80].

### 2.5. Hepatocyte Autophagy and Apoptosis in NAFLD

Damaged organelles or proteins are usually removed by autophagy [60,81,82]. To do so, they are included in the autophagosome, a vacuolar structure which later merges to lysosomes (i.e., autolysosomes), where they are degraded. This catabolic process is aimed to preserve cellular homeostasis by removing non-functional structures and repurposing the product of their degradation inside the cell [83]. Autophagy also plays a role in the mobilization of FFAs from lipid droplets after starvation [84,85,86]; by contrast, an abnormal increase in intracellular lipid could impair autophagic clearance in hepatocytes [84]. This reverse relationship could contribute to the development of a negative loop in which decreased autophagy promotes lipid accumulation that then further suppresses autophagic function, additionally increasing lipid retention [84,87,88,89,90,91,92,93]. Reduced autophagic function could also take part in the accumulation of MDBs in hepatocytes, perpetrating ER stress [83,94,95]. Interestingly, long-term insulin resistance can impair autophagy by reduced expression of transcriptional factors related to autophagic pathways; at the same time, reduced autophagy leads to an increased oxidative damage of the cell, for example by reduced clearance of non-functional mitochondria and increased expression of JNK pathway elements, thus further participating to the vicious cycle that perpetrates pathological processes in the cell [96,97].

The accumulation of different cellular stressors leads to the progression from a state of sublethal injury to, eventually, cellular death [22,24]. Controlled cell death (i.e., apoptosis) is a cellular process aimed to eliminate altered cells in order to preserve the integrity of the tissue; extrinsic (Fas/perforin-mediated) or intrinsic (e.g., ER stress) signaling can reach the mitochondria, releasing cytochrome c into the cytoplasm and leading to cleavage (and subsequent activation) of the protease family of caspases, with terminal apoptosis induction [24,98,99,100,101,102,103]. In NAFLD, multiple intracellular signaling pathways have been proved to trigger apoptosis in hepatocytes (for a detailed review on this topic, see Kanda et al. [104]). Accordingly, when progressing towards NASH, hepatocytes increasingly undergo cell cycle arrest and express apoptosis markers such as caspases and Fas receptors [102,105,106,107,108,109,110]. Interestingly, ballooned hepatocytes represent “undead” hepatocytes, characterized by resistance to apoptotic injury; this is due to a reduced expression of caspases in a Hedgehog-mediated signaling which, however, leads to the activation of pro-inflammatory and pro-fibrogenetic pathways [22,111,112,113,114,115]. In this context, uncontrolled cell death (i.e., necrosis) can occur as disease progresses; this type of cellular death is characterized by cellular damage with release of DAMPs, leading to damage to neighboring cells, to an inflammatory response in immune cells, and to pro-fibrogenetic loops [25,98].

In summary (Table 1), the chronic hepatocellular damage occurring in NAFLD leads to a severe impairment of the cellular mechanisms that are responsible for the clearance of unhealthy and dysfunctional cells; this triggers a tissue response that involves the other cell populations within the liver, and which will be described in the following sections.

## 3. Hepatic Stem/progenitor Cells (HpSCs)

### 3.1. HpSCs are Involved in the Liver Regenerative Response

Hepatic Stem/progenitor Cells (HpSCs) are bipotent progenitor cells, capable to differentiate into mature hepatocytes and cholangiocytes [116,117]. HpSCs are characterized by small size, scant cytoplasm, and an oval nucleus; in liver samples, they can be uniquely individuated by their expression of biliary cytokeratins (e.g., CK7/19) and conventional stem cell markers (e.g., Sox9, CD44, CD133, Epithelial Cell Adhesion Molecule—EpCAM, and Neural Cell Adhesion Molecule—NCAM) [118,119]. HpSCs are facultative stem cells, which are quiescent during physiological turnover of the organ but are activated in acute and chronic liver injuries [120]. HpSCs respond to various stimuli and, once activated, they generate a peculiar morphological tissue response characterized by the appearance of the so-called ductular reaction (DR) [121,122,123]. DR is constituted of reactive ductules, twisting strings of CK7/19^+^ cells without a distinct lumen, and it can show a heterogeneous and highly variable phenotype, which is influenced by the regenerative needs due to the specific disease etiology [119,124].

The actual contribution of the HpSC niche to the renewal of liver parenchyma is at the center of active debate in the scientific community. Using different lineage tracing approaches, it has been observed only a marginal contribution of HpSC in several models of hepatocellular injury [125,126,127]. However, other eminent studies indicated this biliary epithelial compartment as an important source of newly-formed hepatocytes in models where mature hepatocyte proliferation was experimentally impaired [128,129]. Particularly, a progressive HpSC differentiation into mature, functional hepatocytes was observed in genetic mouse models characterized by the induction of apoptosis in 98% of hepatocytes [130] or by the specific blocking of crucial elements of hepatocyte replication pathways [128,129]. Furthermore, elegant models implying long term injury acknowledged the occurrence of DR/HpSC activation as a crucial prerequisite for hepatocyte repopulation [86,131]. Overall, when interpreted together, these evidences indicate that HpSCs represent a quiescent stem cell compartment, which is recruited in course of high-degree and/or long-term liver injury characterized by severe impairment of hepatocyte replicative capabilities and, in the appropriate conditions, can drive a regenerative response allowing liver regeneration.

### 3.2. HpSCs and Their Niche

**Figure 2:**
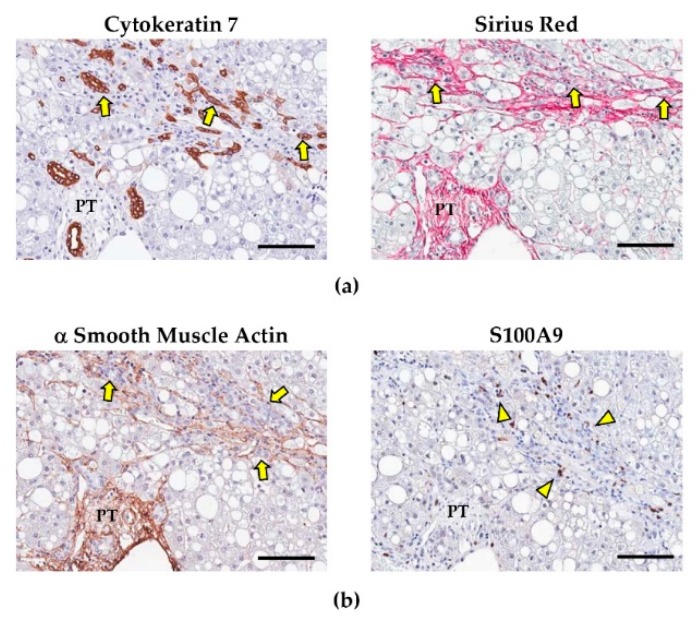
Ductular reaction (DR), myofibroblasts, and portal macrophages in non-alcoholic fatty liver disease (NAFLD). (**a**) As NAFLD progresses from simple steatosis to non-alcoholic steatohepatitis (NASH), a prominent DR emerges (arrows in image on the left) and is associated with portal/periportal fibrosis, as evidenced in Sirius Red stains (arrows in image on the right). (**b**) The expansion of DR is associated with the activation of (α smooth muscle actin-positive) hepatic stellate cells and portal myofibroblasts (arrows), and the recruitment of pro-inflammatory (S100A9^+^) macrophages (arrowheads), which participate in portal/periportal fibrogenetic pathway. PT: portal tract. Scale bars: 100 μm. Images obtained from liver biopsies of patients affected by NAFLD.

HpSCs are supported by a specialized anatomical and functional niche, composed of portal myofibroblasts, hepatic stellate cells (HSCs) and resident macrophages (i.e., Kupffer cells) (Figure 2) [132,133,134]. A crucial function of the niche is the production of several humoral factors, which support HpSC behavior and influence their activation/differentiation state [135]. The main signaling pathways involved in HpSC niche are represented by Notch and WNT systems. HSCs and myofibroblasts can secrete a variety of Notch ligands, which have the role of maintaining HpSCs in a biliary phenotype [119,132,136]. Conversely, the presentation of WNT ligands to HpSCs induces their proliferation and their commitment to the hepatocyte fate [132,135,137]. Macrophages are the main source of WNT ligands within the niche [138,139].

In turn, HpSCs themselves can produce factors that regulate the activation state of non-parenchymal cells within the niche [134]; for instance, HpSC proliferation activates portal myofibroblast/HSC pool by the secretion of Hedgehog ligands, osteopontin (OPN), and transforming growth factor (TGF)-β1 [140]. In liver disease, this can result in the induction of collagen deposition [141,142], leading to fibrogenesis and disease stage progression [121,143].

### 3.3. HpSCs and Their Involvement in NAFLD Progression

**Figure 3:**
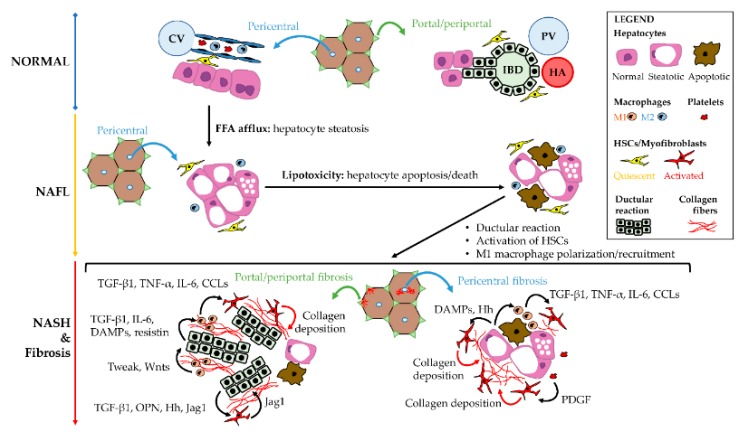
Cellular cross-talks in the pathogenesis of non-alcoholic fatty liver disease (NAFLD). The increase of free-fatty acid (FFA) afflux to the liver determinates hepatocyte steatosis (non-alcoholic fatty liver - NAFL); subsequently, the accumulation of abnormal lipid compounds in the hepatocytes causes lipotoxicity, leading to hepatocyte damage, apoptosis and death. Hepatocyte lipotoxicity triggers M1 macrophage recruitment and lobular inflammation (i.e., steatohepatitis: NASH) and, then, pro-fibrogenetic pathways. In pericentral zone, the activation of hepatic stellate cells (HSCs) and the M1 macrophage polarization trigger perisinusoidal fibrosis. At periportal location, ductular reaction emerges and drives the activation of local myofibroblast pools together with M1 macrophage recruitment. The main molecular factors implied in local cellular cross-talks are summarized in the scheme.

In NAFLD, DR has been extensively studied and it has been correlated with the severity of damage and the progression of liver disease (Figure 3). In these patients, a prominent DR characterizes both adult [144] and pediatric [110] populations affected by advanced stages (i.e., NASH and NASH-fibrosis). Interestingly, DR extent has been correlated with hepatocyte apoptosis, cell cycle arrest, and oxidative stress; thus, indicating that HpSC activation is triggered by progressive hepatocyte cell injury [110]; moreover, in NAFLD, DR is associated with the emergence from reactive ductules of cells with signs of hepatocyte differentiation [110,144].

Remarkably, there is a strict correlation between DR extension and the entity of portal fibrosis and portal inflammation [110,144,145,146]. This correlation is due to the cross-talks between HpSC and non-parenchymal cells (i.e., myofibroblasts and macrophages) within the liver [134], as further discussed later in this review (Figure 3). The activation of HpSC niche could have a significant role in influencing the clinical spectrum of NAFLD, independently to the severity of hepatocyte damage [44]. In NAFLD, pediatric patients also suffering from obstructive sleep apnea syndrome are characterized by higher activation of HpSC niche, with nocturnal hypoxemia being an independent predictor of HpSC activation [147]. Moreover, a peculiar HpSC activation pattern can be observed in patients carrying PNPLA3 I148M variant; the presence of PNPLA3 variant was associated with a more prominent DR and recruitment of cellular components of the niche (i.e., activated myofibroblasts and pro-inflammatory macrophages), independently to the disease grade and stage [44].

## 4. Non-Parenchymal Cells: Supporting the HpSC Response in NAFLD

### 4.1. Hepatic Stellate Cells and Portal Myofibroblasts: Fibrogenetic Pathways in NAFLD

The source of fibrillar collagen in pathological conditions is represented by HSCs and portal myofibroblasts [148,149]. HSCs are perisinusoidal cells located within the space of Disse. In homeostatic conditions, HSCs are quiescent cells [150] and their main functional role is Vitamin A storage; however, in the course of liver injuries, HSCs can trans-differentiate into activated myofibroblast-like cells [151,152,153].

In normal conditions, the liver is characterized by a unique organization of the extracellular matrix (ECM) within the space of Disse: the cords of hepatocytes that constitute the liver lobule are lining on a discontinuous basal membrane, accompanied by few reticular ECM fibers (e.g., type IV collagens, laminin, and perlecan); differently, fibrillar collagens (mostly type I, III, and V) are mainly located around the portal tract, where they constitute a more dense fibrous network [154,155,156]. However, the tissue response to injury and the activation and trans-differentiation of HSCs lead to a complete remodeling of the ECM, from both a qualitative and a quantitative point of view [156,157]. In particular, the deposition of collagens increases, with a relevant proportion of fibrillar collagens, and ECM proteins such as fibulin-5, vitronectin and lumican [149,157,158,159,160,161]. This becomes even more apparent as disease progresses, as demonstrated by an interesting study of liver transcriptome of NAFLD patients which has revealed the upregulation of genes related to ECM organization in NASH compared to NAFL patients, mediated by the activation of Hedgehog pathway [162].

Traditionally, liver fibrosis (especially in advanced stages) has been considered a “static” condition, with an inevitable progression towards liver cirrhosis. In this context, as NAFLD progresses, the remodeling of fibrotic tissues appears to be impaired due to a reduced intrinsic activity of matrix metalloproteinases (MMPs) and to an increased production of tissue inhibitors of metalloproteinases (TIMPs), with an altered ECM balance that favors the accumulation of pro-fibrogenetic ECM compounds [160,163,164,165]. However, several clinical trials in subjects with NAFLD have shown how the improvement of clinical status is accompanied by an amelioration of histological depicts, including a significant reduction of fibrosis stage [166,167,168,169]. Moreover, a constant remodeling of the fibrous tissues occurs, releasing fragments of ECM proteins (with the collagen III fragment pro-C3 being one of the most validated ones [170,171]), which can be isolated from the serum of NAFLD patients and can help identify, in particular, patients in advanced fibrosis stages [157,159,172,173].

The patterns of liver fibrosis vary according to the specific disease aetiology [121,174]; in chronic viral hepatitis, hepatocyte damage is mostly located in zone 1 within the liver lobule; the consequent piecemeal necrosis triggers periportal HSCs and portal myofibroblasts; thus, determining portal expansion followed by periportal fibrosis, septal (bridging) fibrosis, and cirrhosis [175]. A similar portal/periportal pattern is observed in biliary fibrosis, which is due to bile duct damage and cholestasis, as in primary biliary cholangitis and primary sclerosing cholangitis [132]. Differently, in alcoholic liver disease or in NAFLD, primary damage involves pericentral (i.e., zone 3) hepatocytes, and, thus, fibrosis conventionally starts with a centrilobular/perivenular distribution and perisinusoidal fibrosis. More recently, two distinct patterns of liver fibrosis have been individuated in NAFLD [174]; in pediatric patients with NAFLD, a portal/periportal fibrosis pattern is predominant [110]. In adults, a centrilobular pattern of perisinusoidal fibrosis is typically observed; however, portal/periportal fibrosis is also described [44].

Portal fibrosis has been pathogenically associated to the activation of HpSC niche and DR appearance, since HpSCs can activate fibrogenetic cells by the secretion of numerous signals, including Hedgehog ligands, TGF-β, TNF-like weak inducer of apoptosis (TWEAK), and platelet-derived growth factor (PDGF) [121]. In keeping with that, DR is correlated with fibrosis and HSC activation both in adult and in pediatric patients [110]. Interestingly, adult patients carrying I148M PNPLA3 variant are characterized by the loss of a predominant pericentral pattern of liver damage and fibrosis, which is associated to increased DR extent independently to other clinical and histological parameters [44].

### 4.2. Liver Macrophages and Their Role in Influencing Fibrogenesis and HpSC Response

The liver macrophage pool is composed of heterogeneous populations. Resident macrophages (Kupffer cells: KCs) are located within hepatic sinusoids [176] and, in physiological conditions, are involved in tissue homeostasis, phagocytosis of cellular debris, iron homeostasis and in the modulation of immune response [176]; indeed, KCs regulate dendritic cell and T lymphocyte activation [177,178,179]. On the other hand, infiltrating monocytes can derive from circulating monocytes [176].

In NAFLD, several experimental evidences have indicated that the macrophage pool has a pivotal role in inflammatory processes and in NASH development, with the emergence of pro-inflammatory macrophages (i.e., classically activated macrophages, or M1 polarized). In mouse models, the depletion of KCs determined the marked reduction of hepatic inflammation in NASH [180,181]. Resident KCs can accumulate large amounts of toxic lipids and transform into foam cells [176]; lipid loaded macrophages are committed to a M1 phenotype and are active in the production of pro-inflammatory cytokines such as tumor necrosis factor (TNF)-α [182]. Moreover, M1 macrophages express toll-like receptor 4 (TLR4), which is implicated in intracellular signaling and response to various pathogenetic stimuli such as DAMPs and pathogen-associated molecular patterns (PAMPs), such as lipopolysaccharides (LPS). Binding of ligands to TLR4 induces the activation of nuclear factor (NF)-κB, stimulating cytokine production and proliferation of macrophages [183]. In NAFLD, the activation of TLR4 in macrophages following hepatocyte necrosis and LPS translation within the liver contributes to local inflammation and correlates with disease progression and DR extent [184].

Conversely, in mouse models, the induction of the M2 activation state (i.e., alternatively-activated macrophages) in resident macrophages is associated with impaired M1 response [185]; macrophages on M2 spectrum ranges are able to promote M1 apoptosis by interleukin (IL)-10 secretion; thus, limiting liver injury and NASH progression [185]. In parallel, NASH is characterized by an enhanced recruitment of circulating monocytes to the injured liver, sustained by KC-derived cytokines; recruited cells further increase the M1 macrophage pool within the liver [176] with a reduction in the M2 compartment [184]. Interestingly, portal infiltration of macrophages seems to be an early event in human NAFLD and predicts progressive disease [145]. Among cytokines, chemokine (C-C motif) ligand 2 (CCL2, or monocyte chemotactic protein 1) mainly contributes to the recruitment of circulating monocytes into the inflamed liver, and its inhibition can impair monocyte recruitment and prevent NASH progression [186,187,188]. In humans, an increased number of CD68^+^ KCs was observed in biopsy samples of patients with more severe NAFLD [183,184]. In children with NAFLD, the number of macrophages increased both in lobular and portal zones; in parallel, a progressive switch to a M1 activation state was observed, in correlation with disease stage [137]. Portal infiltration of macrophages also seems to be an early event in human NAFLD and predict progressive disease [145].

Liver macrophage pool orchestrates several interactions and cross-talks among resident or recruited cells; thus, driving inflammatory processes and fibrogenesis during the progression of NAFLD [189]. The spectrum of liver macrophage activation is also relevant for fibrosis progression in NAFLD. Liver macrophage on the M1 spectrum ranges could trigger HSC trans-differentiation, and their depletion in mouse models attenuates the fibrosis progression [189]. From a molecular point of view, macrophages can i) activate HSCs by releasing TGF-β and other pro-fibrogenetic cytokines, ii) promote HSC survival and TIMP-1 production via TNF-α and IL-1 secretion [190,191], and iii) promote HSC migration via the secretion of CCL2, CCL3-5, CCL7, and CCL8 [192].

Liver macrophages can have a role in regulating liver regeneration by influencing HpSCs niche [193]. Among the variety of cytokines produced by macrophages, TWEAK is a potent mitogen for HpSCs [138,139]. Furthermore, macrophages are able to secrete WNT ligands (e.g., Wnt3a), thus activating canonical Wnt pathway in HpSCs and triggering their commitment towards hepatocyte fate [135,137]. The Wnt3a production by macrophages is determined by an efficient phagocytosis of the hepatocyte debris [135]. In turn, proliferating HpSCs could secrete a variety of compounds which influence macrophage activation state [141,142]. Indeed, adipocytokines (i.e., adipose tissue cytokines) could represent an intriguing tool in the cellular cross-talks among HpSCs and liver macrophages [110]. In pediatric NASH, HpSCs down-regulate their adiponectin production and, on the other hand, up-regulated their expression of resistin in correlation with progression towards NASH and fibrosis [194]. Adiponectin exerts anti-inflammatory properties and is able to ameliorate inflammation when administered in experimental NASH [195,196]. By contrast, resistin is a mediator of hepatic inflammation and macrophage activation and its administration in rats significantly worsens inflammation [197] by increasing macrophage recruitment and proinflammatory cytokine expression [195,197].

### 4.3. Re-Shaping HpSC Niche as a Therapeutic Approach in NAFLD Patients

Therapies able to improve liver histology in NAFLD patients have also a significant effect on the HpSC niche, supporting its role in disease progression.

In a clinical trial on pediatric patients with NAFLD, the administration of docosahexaenoic acid (a polyunsaturated fatty acid) has been proved to improve liver steatosis and insulin sensitivity. In parallel, docosahexaenoic acid administration determined a re-shaping of HpSC niche by also modulating macrophage activation states [137,169,198]. Remarkably, docosahexaenoic acid treatment determined a reduction in HpSC number and a macrophage polarization towards an anti-inflammatory (M2) phenotype; these changes correlated with amelioration in liver histology. Furthermore, macrophage polarization state towards M2 was correlated with the reduction of serum inflammatory cytokines, with increased macrophage apoptosis, and with the up-regulation of macrophage Wnt3a expression; in turn, macrophage Wnt3a expression was correlated with β-catenin phosphorylation in hepatic progenitor cells and signs of commitment towards hepatocyte fate. 

Interestingly, the combined therapy with docosahexaenoic acid and vitamin D in pediatric NAFLD patients lead to the reduction in myofibroblast activation and fibrogenesis in correlation with histological depicts [169]. Finally, in obese patients affected by NAFLD, laparoscopic sleeve gastrectomy has been proved to determine the amelioration in NAFLD disease stage and grade; this improvement was associated with the reduction of hepatocyte senescence, HpSC activation and recruitment of cellular components of the niche [166].

In sum (Table 2), HpSC niche activation represents a key factor in the local response to injury in NAFLD patients, actively participating in modulating inflammation and fibrogenetic processes. The development of integrated therapies for NAFLD/NASH should consider the signaling pathways acting in HpSC niche, in order to achieve the optimal tissue remodeling that is required to prevent disease progression.

## 5. Interaction of Liver Cellular Compartments with Extra-Hepatic Organs

**Figure 4:**
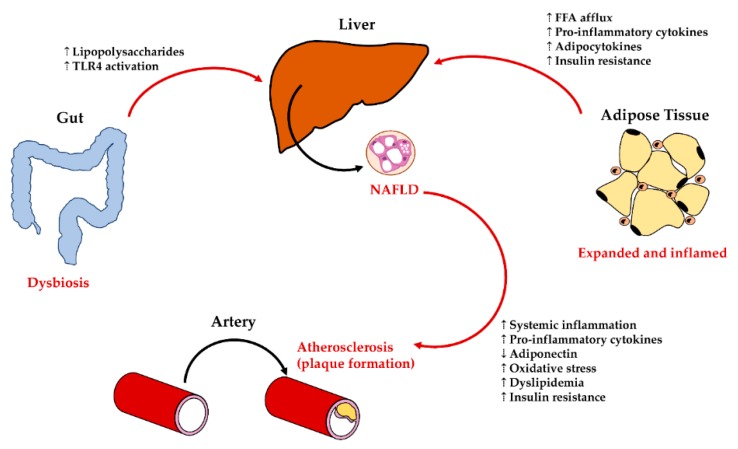
Interaction of liver damage with extra-hepatic organs. NAFLD is influences by interaction with other organs/tissues. Adipose tissue disarrangement (expansion/inflammation) induces increased Free Fatty Acid (FFA) afflux to the liver and insulin resistance; moreover, it releases several pro-inflammatory cytokines and modifies the adipocytokine balance. Dysbiosis in the gut results in translocation of endotoxins (i.e., lipopolysaccharides) to the liver and the subsequent activation of the Toll-like Receptor (TLR) pathway in the liver. In turn, liver with NAFLD/NASH can influence atherosclerosis (plaque formation) by several mechanisms, including, but not limited to, systemic inflammation and oxidative stress increase.

The clinical management of NAFLD patients has demonstrated how this disease should be considered in a broader scenario and how patients should be framed with a multi-disciplinary approach, given the mutual influence between NAFLD and the other organ diseases (e.g., heart failure, atherosclerosis, arterial hypertension, diabetes, chronic kidney disease, gut dysbiosis, obesity, and metabolic syndrome) [6]. Although these clinical manifestations are now well recognized in NAFLD and led to changes in international guidelines recommendation for patient management [1], the mechanisms of these systemic interactions are less known, both at cellular and molecular levels. However, it is now evident that factors coming from the gut (i.e., bacterial translocation) and from the adipose tissue (i.e., adipocytokines) could interact with both parenchymal and non-parenchymal liver cell populations; in turn, liver inflammation, hepatic insulin resistance, and local oxidative stress can affect other organs. This section aims to summarize the most relevant interactions between liver cells and extra-hepatic organs contributing to NAFLD progression (Figure 4).

### 5.1. Liver—Adipose Tissue Axis: Influences on Liver Cells in NAFLD

The adipose tissue is considered an immuno-metabolic organ, able to store free fatty acids (FFAs) and maintain the metabolic rate [199]. In particular, visceral adipose tissue is also characterized by the secretion of regulatory cytokines (i.e., adipocytokines) [200,201]. The term adipocytokines include a variety of peptides primarily identified in the adipose tissue and produced by adipocytes (e.g., adiponectin, resistin, leptin) or by local macrophages (e.g., TNF-α, IL-6), which play a role in modulating insulin resistance and inflammatory responses [200]. Obesity is characterized by the excessive accumulation of lipids in the adipose tissue, which promotes the development of insulin resistance and sustains a chronic pro-inflammatory state within adipose tissue [202,203].

Progressive adipose tissue dysfunction and insulin resistance represent key events in NASH development, supporting the existence of an adipose tissue–liver crosstalk [183,204]. Adipocyte hypertrophy and fibrosis can induce the shift of FFAs to the liver, contributing to hepatic steatosis and to NAFLD progression [205]. In this context, the increased flux of FFAs to the liver contributes to lipotoxicity in hepatocytes, leading to NASH [206,207]; in keeping, diseased hepatocytes could activate Kupffer cells through pattern recognition receptors (e.g., TLRs) and induce local pro-inflammatory cytokine release. Furthermore, adipose tissue could influence hepatic damage through its secretion of pro-inflammatory cytokines, contributing to low-grade systemic inflammation and insulin resistance [183]. The liver itself has been proven to be a source of adipocytokines [110,137,166,208].

Studies in adult obese subjects suggest that macrophage number in adipose tissue is associated with the severity of hepatic inflammation and fibrosis [209,210,211]. Accordingly, bariatric surgery reduces adipose tissue inflammation and, concomitantly, was shown to determine the improvement of liver histology [166]; this latter is associated with macrophage pool modifications and with a re-shaping of liver and adipose tissue adipocytokine profile [166,212].

### 5.2. Liver—Gut Axis: Influences on Liver Damage in NAFLD

Growing evidence supports an important role for the gut–liver axis in the pathogenesis of NAFLD and NASH [183]. A small intestine bacterial overgrowth contributing to increased serum endotoxemia has been described in NAFLD, with Escherichia Coli being the most abundant bacterium [213]. Experimental studies in animals defined the role of lipopolysaccharides (LPS) from gut microbiota in favoring the occurrence of NASH; the administration of non-lethal doses of endotoxins resulted in FAs accumulation in the liver and steatohepatitis [214]. Moreover, the administration of probiotics or antibiotics in animal models of NAFLD reduced inflammation and liver injury [215].

The mechanistic interplay between LPS and liver cell compartments in subjects affected by NAFLD is less clear. Studies in rodents individuate the LPS–TLR4 signaling as crucial in the gut contribution to NAFLD pathogenesis. Macrophages among other cells are potently activated by endotoxin through the TLR4 pathway. However, after infusion into portal vein, LPS is taken up by hepatocytes and secreted into the bile canalicular system [216,217]; LPS is not fully metabolized by liver cells and it is in fact detected in the human peripheral circulation [218]. A recent study indicates that hepatocyte LPS localization in NAFLD patients is associated to liver histologic damage, LPS engulfment by hepatocytes with impaired ability to LPS clearance as a main trigger of the liver inflammatory process [184]. Furthermore, hepatic LPS content can activate TRL4/NF-κB pathway in local cells, including HpSC, macrophages and platelets, enhancing vicious interactions among resident and recruited cells at the basis of NASH progression [184].

### 5.3. Liver—Cardiovascular System Interplay in NAFLD

The interplay between liver and cardiovascular system has been hypothesized based on the recent evidence in the increased cardiovascular risk in NAFLD patients [6]. 

In multiple large meta-analyses, patients with NAFLD showed a higher risk of cardiovascular disease events than those without NAFLD [219,220,221]. Severity of liver disease (i.e., NASH diagnosis) appeared to be associated with an increase in cardiovascular events [219,220,221]. Moreover, NAFLD was associated to cardiovascular risk factors, as hypertension and atherosclerosis. Particularly, subclinical and clinical atherosclerosis have been associated to NAFLD, independently to other known risk factors [6]. Less is known regarding pathogenetic mechanisms, linking the liver and the cardiovascular diseases.

NAFLD increases the risk of developing cardiovascular disease through numerous proposed pathophysiological mechanisms [6]. As discussed above, NAFLD induces systemic inflammation, hepatic insulin resistance, lipid metabolism alteration, and oxidative stress; the inflamed liver is a source of proinflammatory cytokines and adipocytokines, produced by diseased hepatocytes, HpSCs, and M1-polarized Kupffer cells [222]. Systemic inflammation induces endothelial dysfunction, alters vascular tone, and enhances vascular plaque formation [222]. Hepatic lobular inflammation, independently from steatosis, can alter serum lipid profiles, causing abnormally elevated TG, VLDL, and LDL levels, as well as abnormally decreased HDL levels [223]. Finally, hepatocyte alterations in NAFLD are responsible for insulin resistance and contribute to systemic oxidative stress, which are a risk factor for cardiovascular diseases [44,222,224].

## 6. Conclusions

NAFLD is a chronic liver disease and its global prevalence is constantly increasing. The individuation of drugs for NAFLD represents a current effort for clinical researchers. The individuation of cellular and molecular cross-talks between resident liver cells is crucial to define the progression toward steatohepatitis and fibrosis, conditions that are linked to a worse disease evolution and clinical prognosis. Moreover, NAFLD is associated with several alterations in other systems and organs, including the cardiovascular system, digestive tract organs, and adipose tissue, as well as metabolic and endocrine homeostasis. Therefore, the study of interaction between the liver and other organs is important for a systemic approach to NAFLD, and crucial—not only from a clinical—but also from a pathogenetic point of view. In this scenario, therapeutic/pharmacological strategies to prevent fibrosis progression requires the individuation of targetable pathways and adequate models that take into account the cellular and humoral microenvironment at the basis of disease progression.

## Figures and Tables

**Table 1:** Modifications in hepatocytes in NAFLD.

NON-ALCOHOLIC FATTY LIVER	NON-ALCOHOLIC STEATOHEPATITIS
**Hepatic steatosis** Increased fat intake Insulin resistance Lipolysis from adipose tissue De novo lipogenesis Lipopolysaccharides (LPS) localization (low)	**Lipotoxicity** Hepatocellular ballooning Endoplasmic reticulum stress Mitochondrial alterations Oxidative stress Damaged organelles/proteins Hepatocyte apoptosis/necrosis LPS localization (high)

**Table 2:** Phenotypical changes within Hepatic Stem/progenitor Cell niche in NAFLD.

	NON-ALCOHOLIC FATTY LIVER	NON-ALCOHOLIC STEATOHEPATITIS
**Hepatic stem/progenitor cells**	**Mostly quiescent**	**Activation** Ductular reaction Cytokine release Signaling molecule release
**Hepatic stellate cells & portal myofibroblast pool**	**Mostly quiescent** Reticular extracellular matrix (ECM) production Initial perisinusoidal fibrosis	**Activation** Fibrillar ECM proteins Progressive fibrosis Signaling molecule release
**Liver macrophage pool**	**Lobular macrophages** ↑ Lobular macrophages ↓ Lobular M2 macrophages **Portal macrophages** No modifications	**Lobular macrophages** ↑ M1 lobular macrophages **Portal macrophages** ↑ Portal macrophages ↑ M1 portal macrophages ↓ M2 portal macrophages
